# Dimerization Tendency of 3CLpros of Human Coronaviruses Based on the X-ray Crystal Structure of the Catalytic Domain of SARS-CoV-2 3CLpro

**DOI:** 10.3390/ijms23095268

**Published:** 2022-05-09

**Authors:** Seri Jo, Hwa Young Kim, Dong Hae Shin, Mi-Sun Kim

**Affiliations:** College of Pharmacy and Graduates School of Pharmaceutical Sciences, Ewha Womans University, Seoul 03760, Korea; seri9388@gmail.com (S.J.); vkwm0221@naver.com (H.Y.K.)

**Keywords:** SARS-CoV-2 3CL protease, catalytic domain, X-ray crystallography

## Abstract

3CLpro of SARS-CoV-2 is a promising target for developing anti-COVID19 agents. In order to evaluate the catalytic activity of 3CLpros according to the presence or absence of the dimerization domain, two forms had been purified and tested. Enzyme kinetic studies with a FRET method revealed that the catalytic domain alone presents enzymatic activity, despite it being approximately 8.6 times less than that in the full domain. The catalytic domain was crystallized and its X-ray crystal structure has been determined to 2.3 Å resolution. There are four protomers in the asymmetric unit. Intriguingly, they were packed as a dimer though the dimerization domain was absent. The RMSD of superimposed two catalytic domains was 0.190 for 182 Cα atoms. A part of the long hinge loop (LH-loop) from Gln189 to Asp197 was not built in the model due to its flexibility. The crystal structure indicates that the decreased proteolytic activity of the catalytic domain was due to the incomplete construction of the substrate binding part built by the LH-loop. A structural survey with other 3CLpros showed that SARS-CoV families do not have interactions between DM-loop due to the conformational difference at the last turn of helix α7 compared with others. Therefore, we can conclude that the monomeric form contains nascent enzyme activity and that its efficiency increases by dimerization. This new insight may contribute to understanding the behavior of SARS-CoV-2 3CLpro and thus be useful in developing anti-COVID-19 agents.

## 1. Introduction

Severe acute respiratory syndrome (SARS-CoV-2), which emerged in Wuhan, China in December 2019, has now spread worldwide [1,2], with more than 480 million people having been infected and about 6 million having died as of 5 April 2022 (https://covid19.who.int/ accessed on 5 April 2022). The virus is the seventh coronavirus that has infected humans and the third β-coronavirus to cause an outbreak in the 21st century, following the Severe Acute Respiratory Syndrome (SARS-CoV) of 2003 and the Middle East Respiratory Syndrome (MERS) of 2012 [3,4,5]. A wide range of diseases have been reported, from mild to severe diseases, including death [6,7,8]. The incubation period of 5 to 6 days and the SARS-CoV-2 reproduction rate of 2.2 to 3.6 days have accelerated the spread of COVID-19 [9,10]. To date, there are several FDA-approved vaccines for SARS-CoV-2, but there is still no strong and effective treatment for COVID-19. Although Remdesivir has been scientifically found to be helpful in treating COVID-19, it is not a particularly effective drug. Currently, among the chemicals, paxlovid, molnupiravir, and remdesivir have been granted emergency use permission for the treatment of patients hospitalized with COVID-19 from the U.S. Food and Drug Administration (FDA) [11,12,13].

Several X-ray crystal structures of SARS-CoV-2 main protein enzymes (3CLpro or Mpro) combined with lead compounds serving as inhibitors have recently been announced [14,15,16]. The 3CLpro is a cysteine protease that plays an essential role in the replication of coronaviruses by digesting viral polyprotein at more than 11 sites. Therefore, it is considered and selected as a good COVID-19 target. In addition, the 3CLpro is almost preserved among all released SARS coronavirus genome sequences and is very homogeneous with other coronavirus 3CLpros. Specifically, the sequence identity of 3CLpros between SARS-CoV-2 and SARS-CoV is 96%, and only one different residue is present at the active site. Structurally, 3CLpros of SARS-CoVs include the following three domains. Domains I and II (residues 1 to 184) have an antiparallel β-barrel structure that forms chymotrypsin folds, and the substrate binding site is located in the gap between these two domains. Additionally, domain III (residues 201 to 303) is a compact α-helical domain connected to domain II by a long hinge loop (LH-loop, residues from Gln189 to Asp197) [16,17]. The protease is known to be activated by dimerization of two monomers, each containing a catalyst dyad defined by Cys145 and His41 residues (active site) [18]. An important role of domain III in the dimerization and catalytic activity of 3CLpro has been suggested by several studies [19,20].

Though X-ray crystal structures of the entire enzyme have been published, some of structural and catalytic roles of domain III are still unclear. For example, since the dimeric interfaces of domain III in the crystal structure is quite narrow, its role in dimerization seems limited. In addition, its contribution in the catalytic mechanism is also not well explained. In this study, we determined the X-ray crystal structure of the catalytic domain of SARS-CoV-2 3CLpro and performed fluorescence resonance energy transfer (FRET)-based enzymatic assays against entire and catalytic domains. A new insight for the catalytic domain is discussed in this study.

## 2. Results and Discussion

### 2.1. Structural Properties of the Catalytic Domain of SARS-CoV-2 3CLpro

**Figure 1:**
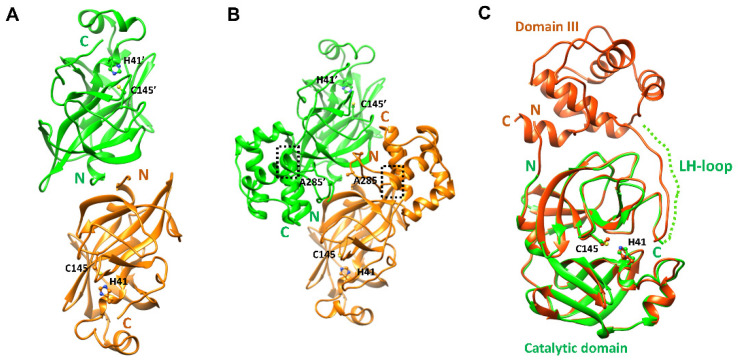
**The X-ray crystal structure of the catalytic domain of SARS-CoV-2 3CLpro compared with the model structure.** Each subunit is colored differently. Active site residues are drawn with a ball-and-stick model. N- and C-terminals are labeled. (**A**) The X-ray crystal structure of the catalytic domain of SARS-CoV-2. (**B**) The crystal structure of the entire SARS-CoV-2 3CLpro (7K0F). Ala285 residue on the DM-loop was depicted. The dotted boxes represent the last turn of helix α7. (**C**) The superposition of both monomeric forms. The LH-loop is displayed with a dotted line.

**Figure 2:**
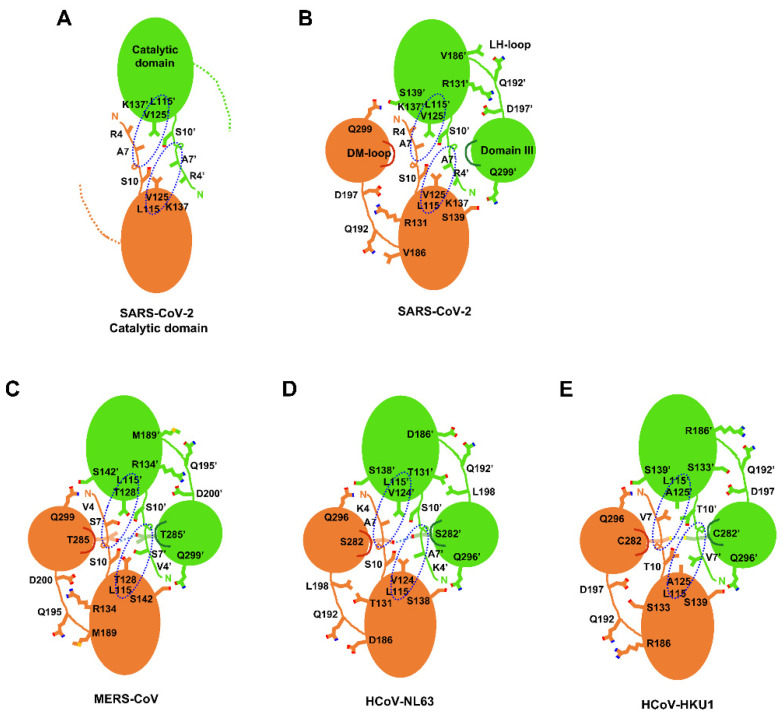
**Key interactions involved in dimerization.** The two subunits were drawn with different colors, and key residues were represented by a stick model. The distances of hydrogen bonds between residues on domain III are found in the text. The dotted blue circles indicate where hydrophobic pockets exit. (**A**) the catalytic domain of SARS-CoV-2 3CLpro; (**B**) SARS-CoV-2 3CLpro; (**C**) MERS- CoV-2 3CLpro; (**D**) HCoV-NL63 3CLpro; (**E**) HCoV-HKU1 3CLpro.

The crystal structure of the catalytic domain of SARS-CoV-2 CLpro was determined at 2.27 Å resolution. In the final model, 182 residues were included. The first six residues (Ser1 and Met6) and the last eight residues (Gln189 to Thr196) in the LH-loop were not visible in the final model though they were predicted to form a short β-strand in PSIPRED [21]. The overall structure of the other region was well determined (Table 1) except residues from Ser139 to Ser144 and displayed almost the same architecture (Figure 1) as that of the entire SARS-CoV-2 CLpro [22,23]. The five residues not well defined were due to the absence of domain III influencing a local conformation around Ser139. As a result, the electron density around Ser139 to Ser144 was not well defined. There were four protomers in the asymmetric unit. Interestingly, they existed as a dimer with dimensions of ~83 × 48 × 36 Å despite the absence of the dimerization domain (domain III) known to be crucial in dimerization [19]. The root-mean-square deviations (RMSDs) of 182 Cα atoms among them were within the range of 0.190–0.479 Å. The RMSD between the monomeric forms of the catalytic domains of the search model and the final refined model was 0.678. When the dimeric forms of the catalytic domains were compared, the RMSDs were 0.682 for 341 Cα atoms (pruned) and 0.905 for all 364 Cα atoms. Therefore, there was almost no conformational deviation among them. In the crystal structure of the catalytic domain, dimeric interfaces were made by backbone hydrogen pairs of Ala7 N and O atoms with O (3.39 Å) and N (2.88 Å) atoms of Val125’ (the other subunit), respectively (Figure 2A). There were more hydrogen bonds mediated by Ser10 and between Ser11 and Glu14’. A hydrophobic pocket composed of Pro9, Val13, Leu115’, and Val125’ also participated in the formation of a dimer. Therefore, the N-terminal loop contributed to the formation of a domain-swapped dimer by interacting with β10 of the other subunit. In the case of the entire SARS-CoV 3CLpro [24], Arg4 also took part in the interaction with Lys137’ from the catalytic domain of the other subunit (Figure 2B).

**Figure 3:**
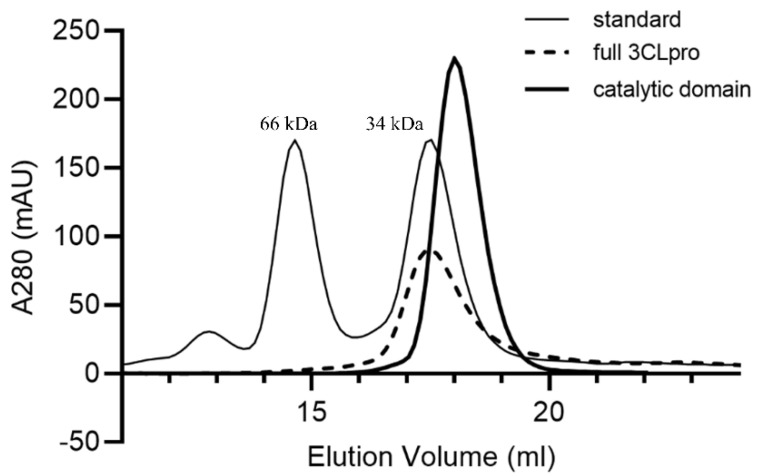
**The size-exclusion chromatography of SARS-CoV-2 3CLpro.** The thin line represents the makers (human albumin and TCTP), the thick line represents the catalytic domain, and the dotted line represents the full 3CLpro.

In order to find the oligomeric state of the catalytic domain in solution, a gel chromatographic experiment was performed. Unexpectedly, only a monomeric form was detected (Figure 3). This implies two facts. First, the dimeric form of the catalytic domain only detected in the X-ray crystallographic study may be a crystallographic artifact. Second, the dimeric interaction mainly caused by the N-terminal swapping is not enough to sustain a stable dimeric architecture in solution. Intriguingly, the entire SARS-CoV-2 3CLpro had an additional dimeric interaction between Ser139 and Gln299’ (2.53 Å) on domain III (Figure 2B) [23]. Mutational experiments of the two residues to alanine clearly demonstrated the transition from the dimeric to monomeric forms with severely reduced catalytic activity [25,26,27]. The importance of the additional bond in the formation of the dimeric state is certified by the sequence conservation in the corresponding positions of 3CLpros (Figure 2) [28]. Briefly, though the N-terminal domain swap is crucial for dimerization, more interactions with domain III may be required to form a stable dimer. A structural comparison of MERS-CoV 3CLpro with SARS-CoV-2 3CLpro indicates that the dimerization of MERS-CoV 3CLpro may be easier due to the interaction between Thr285 and Thr285’ (2.44 Å) on domain III (Figure 2C), as discussed below. This region is far apart due to the absence of the interaction in the case of SARS-CoV-2 3CLpro. It is also worth noting that the presence of monomeric forms of the entire 3CLpro were frequently reported during size-exclusion chromatography (SEC) [29,30,31]. We also performed SEC of the entire domain and detected a monomeric form in Tris and sodium phosphate buffers (Figure 3). This implies that the dimeric state of SARS-CoV-2 3CLpro is not very strong and requires some condition such as the presence of substrates [27,32].

**Figure 4:**
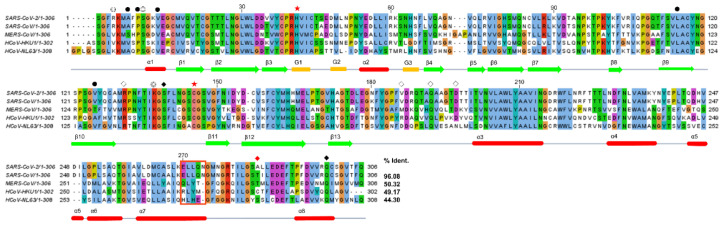
**The sequence alignment of 3CLpros.** Secondary structure elements of 3CLpro are displayed and colored in red for α-helix, in green for β-strand, and in yellow for 3_10_-helix. The sequences of 3CLpros were extracted from PDBs: 7K0F for SARS-CoV-2, 6W2A for SARS-CoV, 4YLU for MERS-CoV, 3D23 for HCoV-HKU1, and 5GWY for HCoV-NL63. The red stars indicate the catalytic residues. The residues involved in the N-terminal loop swapping are depicted with circles. The circles and the filled circles represent hydrophilic and hydrophobic interactions, respectively. The diamonds represent the interaction between the LH-loop and domain III, filled diamonds represent the dimeric interaction between Ser139 and Q299, and red represents the DM-loop interaction.

In the X-ray crystal structure of the catalytic domain, the eight residues included in the LH-loop are not visible in this construct. In contrast, the LH-loop of the entire SARS-CoV-2 3CLpro is well defined due to the following interactions. First, Gln192 interacts with the backbone oxygen and nitrogen atoms of Val186 (Figure 4). Second, the flexible nature of the LH-loop due to the presence of the ^191^AQAAGT^196^ sequence was compensated by the salt-bridge between Arg131 and Asp197. As a result, the entire SARS-CoV-2 3CLpro may exert a full enzyme activity. In addition, Gln189 on the LH-loop takes part in binding with chemicals, as shown in several X-ray crystal structures [33,34]. Therefore, the LH-loop is critical in the structural and functional constancy of SARS-CoV-2 3CLpro by stabilizing an outer wall of the active site cavity together with the substrate binding site (Figure 2).

### 2.2. Enzymatic Properties of the Monomeric Form of the Catalytic Domain

We characterized the enzymatic activity of entire and catalytic domains of SARS-CoV-2 3CLpro by measuring the K_m_ and V_max_ values. When 1 µM 3CLpro was mixed with various concentrations of FRET substrate (0–30 µM), the initial velocity was measured and plotted against the substrate concentration. The curve fitting the Michaelis–Menten equation gave the best-fit values of K_m_ and V_max_ as 5.723 µM and 566.137 RFU/s for the entire domain and as 6.152 µM and 71.135 RFU/s for the catalytic domain. The calculated k_cat_/K_m_ were 712.247 s^−1^ M^−1^ and 83.253 s^−1^ M^−1^, respectively (Supplementary Figure S1). It represents that the enzyme activity of the catalytic domain is about 8.6 times lower than that of the entire domain.

The entire SARS-CoV-2 3CLpro is known to function as a dimer. In addition, its domain III was thought to be crucial for catalytic activity [19]. Nevertheless, there are some significant features of the catalytic domain noteworthy in structural and functional aspects. At first, the N-terminal domain alone can contribute to forming a dimer through the N-terminal loop swap, as shown in the X-ray crystal structures of the entire and catalytic domains of SARS-CoV-2 3CLpro. Second, the monomeric form alone is enough for its catalytic activity despite its low efficiency. Third, the LH-loop is important for substrate binding and catalytic activity by finalizing the construction of the catalytic pocket. Therefore, the catalytic domain alone can exert its enzymatic activity as we observed. Similar properties were also found in the entire SARS-CoV 3CLpro. In low protein concentrations, the monomer form also displayed an enzymatic activity with low efficiency [19].

### 2.3. Structural Comparison among Various 3CLpros

There are five 3CLpros of which the X-ray crystal structures were determined [17,22,28,35,36]. They share a common architecture, as expected based on sequence identity (Figure 4). The domain swapping of the N-terminal loop is a primary feature of all 3CLpros. It also induces the formation of a small hydrophobic core, as mentioned above. In contrast, there is also a key additional interaction intensifying dimerization in some 3CLpros. The interaction mediated by a loop between α7 and α8 of domain III (DM-loop) plays a key role (Figure 2, Supplementary Figure S2). The loop interaction mediated by Thr285 (2.44 Å), Cys282 (3.82 Å), and Ser282 (3.87 Å) of MERS, HCoV-HKU1, and HCoV-NL63, respectively, seems to strengthen the dimeric state. Since these residues on the DM-loop are located next to the two-fold rotational symmetry operator in space, a stable dimeric form may be augmented. However, the corresponding residues of SARS-CoV and SARS-CoV-2 3CLpros are far apart, with their Cα atom distances being 6.88 Å and 8.00 Å, respectively. Those of MERS, HCoV-HKU1, and HCoV-NL63 are 6.19 Å, 6.58 Å, and 5.05 Å, respectively (Supplementary Table S1). This means that the interaction between the DM-loops of SARS-CoV and SARS-CoV-2 3CLpros is weak. In particular, the last turn of helix α7 is well formed in SARS-CoV and SARS-CoV-2 3CLpros due to the presence of ^270^ELLQN^274^ facilitating α-helix formation (Figure 1B). However, Tyr273, Tyr270, and His270 with bigger aromatic groups are found in the corresponding positions of MERS, HCoV-HKU1, and HCoV-NL63, respectively (Figure 4). Therefore, the last turn of α7 is more relaxed and thus makes the distance between DM-loops a bit closer in the latter three cases. As a result, domain III of 3CLpros of MERS, HCoV-HKU1, and HCoV-NL63 contributes to dimerization better than those of SAR-CoV and SARS-CoV-2.

## 3. Materials and Methods

### 3.1. Protein Expression and Purification of SARS-CoV-2 3CLpro and its Catalytic Domain

The coding sequence of SARS-CoV-2 3C-like proteinase (NCBI Ref. seq. YP_009725301.1) was synthesized chemically by Bioneer, Daejeon, Korea and cloned into a bacteriophage T7-based expression vector. Its catalytic domain was synthesized from M1 to T196 of 3CLpro. The catalytic one was synthesized by adding ENLYFQGGG (TEV protease cleavage site) and the entire enzyme by adding AVLQ (autocleavage site) at the N-terminus. The plasmid DNA was transformed into *E. coli* BL21 (DE3) for protein expression. *E. coli* BL21 (DE3) cells were grown on Luria–Bertani (LB) agar plates containing 150 μg ml^−1^ ampicillin. Several colonies were picked and grown in capped test tubes with 10 mL LB broth containing 150 μg ml^−1^ ampicillin. A cell stock composed of 0.85 mL culture and 0.15 mL glycerol was prepared and frozen at 193 K for use in a large culture. The frozen cell stock was grown in 5 mL LB medium and diluted into 1000 mL fresh LB medium. The culture was incubated at 310 K with shaking until an OD_600_ of 0.6–0.8 was reached. At this point, the expressions of SARS-CoV-2 3CLpro and its catalytic domain were induced using isopropyl-β-d-1-thiogalactopyranoside (IPTG) at a final concentration of 1 mM. The culture was further grown at 310 K for 3 h in a shaking incubator. Cells were harvested by centrifugation at 7650 g (6500 rev min^−1^) for 10 min in a high-speed refrigerated centrifuge at 277 K. The cultured cell paste was resuspended in 25 mL of a buffer consisting of 20 mM Tris pH 7.5, 1 mM phenylmethylsulfonyl fluoride (PMSF), and 10 μg ml^−1^ DNase I. The cell suspension was disrupted using an ultrasonic cell disruptor (Digital Sonifier 450, Branson, MO, USA). Cell debris was pelleted by centrifugation at 24,900× g (15,000 rev min^−1^) for 30 min in a high-speed refrigerated ultra-centrifuge at 277 K.

The entire SARS-CoV-2 3CLpro was purified by affinity chromatography using a 5 mL Hi-Trap His column (GE Healthcare, Piscataway, NJ, USA). The column was equilibrated with a buffer consisting of 20 mM Tris pH 7.5, and the pooled fractions were loaded. The fractions of 3CLpro-His tag were mixed with Human Rhinovirus (HRV) 3C protease-His-tag at a molar ratio of 10:1 to remove the C-terminal His tag. The His-tag cleaved 3CLpro was further purified by affinity chromatography using a 5 mL Hi-Trap His column (GE Healthcare, Piscataway, NJ, USA) and the flow-through was collected. The purified protein was buffer exchanged into 20 mM Bis-Tris pH 7.5 using Vivaspin 20 MWCO 10 kDa (GE Healthcare), a centrifugal device. SDS–PAGE showed one band around 34 kDa (33,796.64 Da), corresponding to the molecular weight of SARS-CoV-2 3CLpro.

The catalytic domain of SARS-CoV-2 3CLpro was purified by affinity chromatography using a 5 mL Hi-Trap His column (GE Healthcare, Piscataway, NJ, USA). The column was equilibrated with a buffer consisting of 20 mM Tris pH 7.5, and the pooled fractions were loaded. The fractions of 3CLpro-His tag were mixed with TEV protease-His-tag at a molar ratio of 10:1 to remove the N-terminal His tag. The His-tag cleaved 3CLpro was further purified by affinity chromatography using a 5 mL Hi-Trap His column (GE Healthcare, Piscataway, NJ, USA), and the flow-through was collected. The purified protein was buffer exchanged into 20 mM Bis-Tris pH 7.5 using Vivaspin 20 MWCO 10 kDa (GE Healthcare), a centrifugal device. SDS–PAGE showed one band around 22 kDa (22,533.70 Da), corresponding to the molecular weight of the catalytic domain of SARS-CoV-2 3CLpro.

### 3.2. Enzymatic Kinetic Studies with SARS-CoV-2 3CLpro

The custom-synthesized fluorogenic substrate, DABCYL-KTSAVLQSGFRKME-EDANS (ANYGEN, Gwangju, Korea), was used as a substrate for the proteolytic assay using the SARS-CoV-2 3CLpro [37]. This substrate contains the nsp4/nsp5 cleavage sequence, GVLQ↓SG [38], and works as a generic peptide substrate for many coronaviruses including the SARS-CoV-2 3CLpro. The peptide was dissolved in distilled water and incubated with each protease. A SpectraMax i3x Multi-mode microplate reader (Molecular Devices) was used to measure spectral-based fluorescence. The proteolytic activity was determined at 310 K by following the increase in fluorescence (λ_excitation_ = 340 nm and λ_emission_ = 490 nm, with bandwidths = 9 and 15 nm, respectively) of EDANS upon peptide hydrolysis as a function of time. The assays were conducted in black, 96-well plates (Nunc) in 300 μL assay buffers containing proteases and substrates, as follows: For the measurements of K_m_/V_max_, a proteolytic reaction with 2 µM 3CLpro in 300 µL of the reaction buffer was carried out at 310 K in a SpectraMax i3x (Molecular Devices) with filters for excitation at 360/40 nm and emission at 460/40 nm. The reaction time was 2 h for both of the SARS-CoV-2 3CLpros at 310 K. A FRET substrate concentration ranging from 0 to 30 µM was applied. The initial velocity was plotted against the FRET concentration with the classic Michaelis–Menten equation in GraphPad Prism 7.03 (GraphPad Software, San Diego, CA, USA). All reactions were carried out in triplicate.

### 3.3. Size-Exlusion Chromatography (SEC)

Size-exclusion chromatography experiments were performed using a 10/30 Superdex 200 column (Amersham Biosciences, Amersham, UK) connected to an ÄKTA-explorer system. The typical flow rate was 0.4 mL min^−1^, fractions of 0.3 mL were collected, and 10 mg mL^−1^ of catalytic domain and 5 mg mg mL^−1^ of the entire domain of SARS-CoV-2 3CLpro were loaded and then eluted with a buffer consisting of 25 mM sodium phosphate (pH 7.0) or 20 mM Tris (pH 7.8) and 100–300 mM NaCl. The column calibration was conducted with 2 proteins: human albumin (65 kDa) and TCTP (34 kDa).

### 3.4. Crystallization

The catalytic domain of SARS-CoV-2 CLpro was crystallized by the hanging drop vapor diffusion method at 296 K with drops consisting of 0.8 µL protein solution (10 mg/mL) and 0.8 µL reservoir solution (10% PEG 20000, 0.1 M HEPES pH 7.0, and 2 M calcium chloride). These initial crystals were used to make a seed stock using the Glass Seed Bead™ kit (Hampton Research, Aliso Vieja, CA, USA). The seeds were prepared from crystals grown in the final crystallization condition. The drop ratios were 0.3 µL protein, 0.2 µL reservoir solution, and 0.1 µL a seed stock dilution of 1/10. Crystals were grown using the hanging drop vapor diffusion method and appeared within 24 h.

### 3.5. Structure Determination

X-ray diffraction data, collected at a single wavelength on the 7 A beamline at the Pohang Light Source, were processed and scaled using the HKL2000 suite [39].

Crystallographic phases for the 2.27 Å catalytic domain of SARS-CoV CLpro dataset were obtained by molecular replacement using the AutoMR wizard in PHENIX [40]. Based on the Matthews coefficient [41], the asymmetric unit could contain four protomers with a solvent content of 48.13% (V_M_  =  2.37 Å^3^ Da^−1^). Iterative density modification, model building, and refinement were performed using the AutoBuild wizard of PHENIX. An electron density map was calculated, and an initial model containing four protomers in the unit cell was obtained. Noncrystallographic symmetry (NCS) matrices were obtained for four protomer molecules in the asymmetric unit and were applied during initial refinement. NCS was released earlier to avoid model bias from the NCS average. The model was further refined using COOT [42] and PHENIX [40].

The final model of the catalytic domain was refined to R_work-_ and R_free-_ values of 20.16% and 26.41%, respectively. The stereochemical quality of the final model was evaluated by PROCHECK [43]. The Ramachandran plot produced with PROCHECK showed 92.78% residues in most favored regions and 6.94% in additional allowed regions. The details of the refinement statistics are listed in Table 1.

## 4. Conclusions

The recent pandemic caused by SARS-CoV-2 is slowly settling down. Nevertheless, the occurrence of mutants is still a threat globally. In this study, structural and enzymatic characterization of the catalytic domain of SARS-CoV-2 3CLpro was provided. Intriguingly, the crystal structure of the catalytic domain showed that the major dimeric interface is formed in the unit cell despite the absence of the dimerization domain, domain III. Therefore, the importance of the N-terminal and LH loops was revisited. The N-terminal loop is critical in the formation of a domain-swapped dimer by contributing to the formation of a hydrophobic cavity with the catalytic domain of the other subunit. Surprisingly, the monomeric form of the catalytic domain alone is proven to be enough for its catalytic activity with low efficiency. Sequence and structural analyses showed that the dimerization tendency of 3CLpors is weaker in SARS species. The relatively weak interaction exerted by the DM-loops between domain IIIs seems to be the main reason. Currently, 3CLpro is a good target for the design of anti-SARS-CoV-2 drugs. Some targeting SARS-CoV-2 3CLpro have already been commercialized: paxlovid, molnupiravir, and remdesivir [11,12,13]. More detailed information of the catalytic domain provided in this study may be helpful in developing better anti-viral agents to cope with COVID-19.

## Figures and Tables

**Table 1:** Data collection statistics and refinement parameters for the catalytic domain of SARS-CoV-2 3CLpro.

PDB Code	7WHC
**Data collection statistics**	
Wavelength	1.0000 Å
Resolution	50.0–2.27 (2.31–2.27) Å
Redundancy	3.4 (3.1) *
Unique reflections	32201 (1203)
Completeness	89.0 (63.2) %
I/σ	17.03 (1.67)
Rmergy ^†^	5.7 (63.2) %
**Crystal parameters and refinement statistics**	
Resolution	31.99–2.27 (2.31–2.27) Å
Space group	P2_1_
Cell dimensions	52.47 Å × 121.09 Å × 62.70 Å
Volume fraction of solvent	48.13%
V_m_ (Å^3^/Dalton)	2.37
Total number of residues	5628
Total non-H atoms	5778
Number of water molecules	150
Average temperature factors	
Protein	48.90 Å^2^
Solvent	45.70 Å^2^
R-factor	20.16%
Free R-factor	26.41%
Stereochemical ideality:	
Bond	0.009 Å
Angle	1.297°
Chirality	0.049°
Planarity	0.006°
Dihedral	13.954°
**Ramachandran plot**	
Residues in favored regions	92.78%
Residues in allowed regions	6.94%
Residues in disallowed regions	0.0%

^†^ R_mergy_ = ∑_hkl_ ∑_i_∣I_hkl, i_ − <I>_hkl_∣/∑_hkl_ ∑_i_ I_hkl, i_, where I_hkl, i_ is the intensity of the ith observation of the unique reflection hkl and <I>_hkl_ is the mean of the intensities of all observations of reflection hkl. * The data were obtained from a single crystal. Values in parentheses are for the highest-resolution shell.

## Data Availability

Not applicable.

## Supplementary Materials

The following supporting information can be downloaded at: https://www.mdpi.com/article/10.3390/ijms23095268/s1.
