# Supplementary material for: Dimerization Tendency of 3CLpros of Human Coronaviruses Based on the X-ray Crystal Structure of the Catalytic Domain of SARS-CoV-2 3CLpro
Source: Int J Mol Sci. 2022 May 9;23(9):5268. doi: 10.3390/ijms23095268 (PMC9103169; doi:10.3390/ijms23095268)
Supplement: Supplementary file 1 [file ijms-23-05268-s001.zip › ijms-1691066-supplementary.pdf]

Supporting Information For

Dimerization tendency of 3CLpros of human coronaviruses based on the X-ray crystal structure of the catalytic domain of SARS-CoV-2 3CLpro

Seri Jo, Hwa young Kim, Dong Hae Shin* and Mi-Sun Kim*
College of Pharmacy, Ewha W. University, Seoul, Republic of Korea 03760

***Co-corresponding authors:**

Dr. Mi-Sun Kim
Department of Pharmacy, Ewha W. University, Seoul, Republic of Korea 03760
Tel: (82) 2-3277-4918; Fax: (82) 2-3277-2851; E-mail: shfwk31@ewha.ac.kr
ORCID: 0000-0002-4092-4203

Prof. Dong Hae Shin
Department of Pharmacy, Ewha W. University, Seoul, Republic of Korea 03760
Tel: (82) 2-3277-4502; Fax: (82) 2-3277-2851; E-mail: dhshin55@ewha.ac.kr
ORCID: 0000-0002-2205-1453

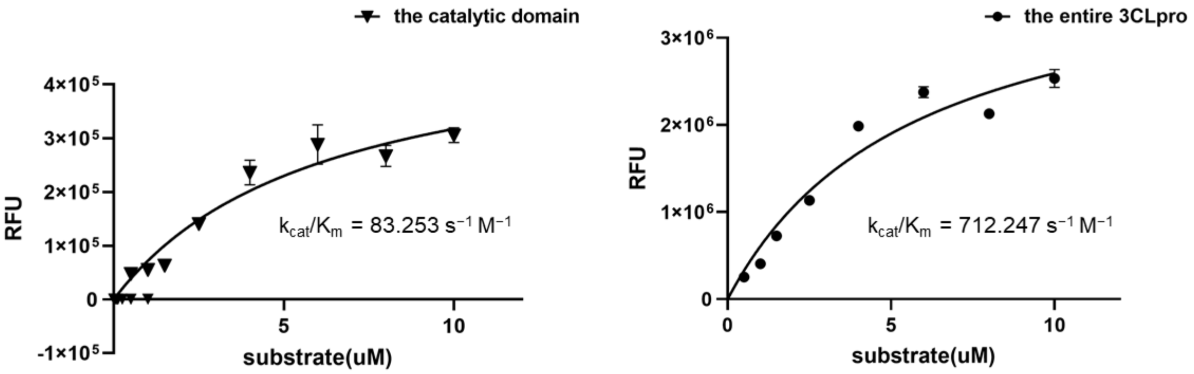

Figure S1. Comparison of catalytic efficiency of the catalytic domain and the entire 3CLpro of SARS-CoV-2. In the kinetic studies, 1 μM 3CLpro was mixed with various concentrations of FRET substrate (0–30 μM). The initial velocity was plotted against the FRET concentration with the classic Michaelis–Menten equation in GraphPad Prism 7.03 (GraphPad Software, San Diego, CA, USA). All reactions were carried out in triplicate. Detailed methods were described in “Materials and methods” section.

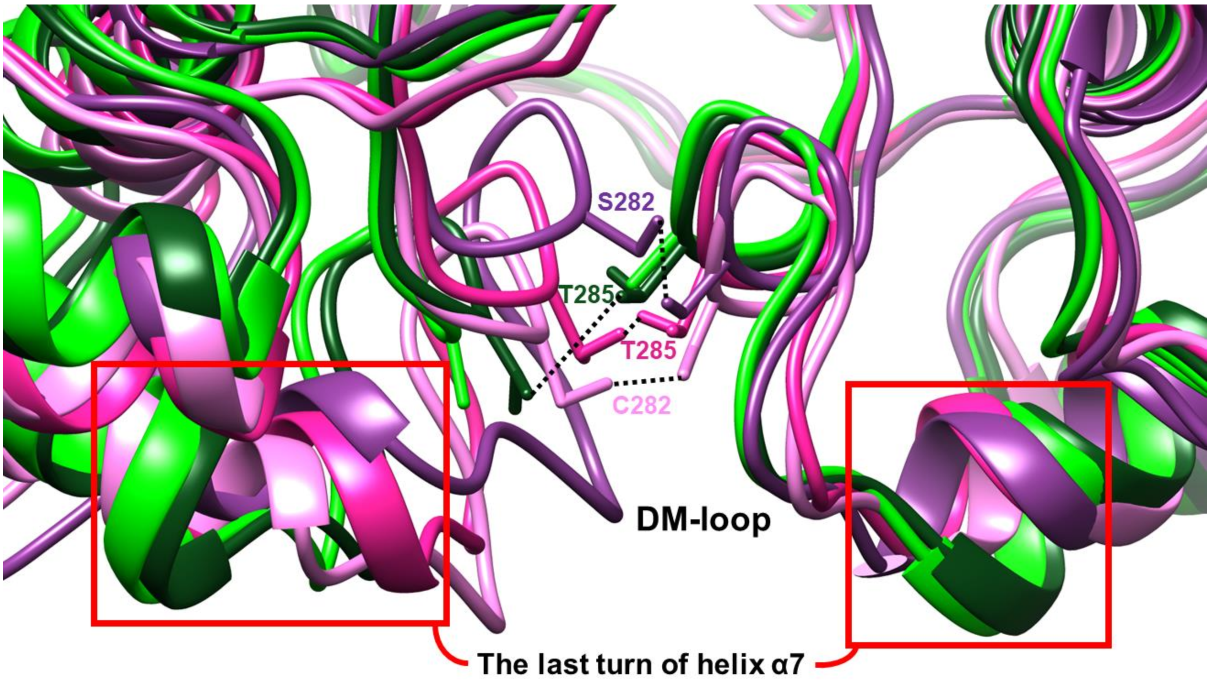

Figure S2. Interaction between DM-loops. The region around DM-loops was drawn. Since domain III of 3CLpro (SARS-CoV-2 (7k0f, bright green), SARS-CoV (6w2a, dark green), MERS-CoV (4ylu, deep pink), HCoV-HKU1 (3d23, light pink), HCoV-NL63 (5gwy, purple)) is linked to the catalytic domain by the LH-loop, its relative position to the other domain III in a dimeric form is varied due to a rigidbody motion even in the crystal structure of the same species. Therefore, the alignment was done only with one subunit and compared with the other subunits. The residues on the DM-loop involved in hydrogen bond formation are labeled. In case of SARS-CoV-2 3CLpro, the corresponding residue is alanine and thus there is no hydrogen bond formation. The last turn of helix α7 are depicted in the red boxes.

Table S1. The residues involved in interaction between the DM-loops.

	SARS-CoV-2	SARS-CoV	MERS-CoV	HCoV-HKU1	HCoV-NL63
**DM-loop interaction**	X	T285 (4.14 Å)	T285 (2.44 Å)	C282 (3.82 Å)	S282 (3.87 Å)
**Cα atom distances (Å)**	6.88	8.00	6.19	6.58	5.05
